# Multiple sclerosis and drug discovery: A work of translation

**DOI:** 10.1016/j.ebiom.2021.103392

**Published:** 2021-05-24

**Authors:** Bert A. ’t Hart, Antonio Luchicchi, Geert J Schenk, Joep Killestein, Jeroen J.G. Geurts

**Affiliations:** a Department Anatomy and Neuroscience, Amsterdam University Medical Center (VUmc), Amsterdam, the Netherlands; b Department Biomedical Sciences of Cells & Systems, University Medical Center, University of Groningen, Groningen, the Netherlands; c MS-center Amsterdam (www.vumc.com), the Netherlands; d Department Neurology, Amsterdam University Medical Center (VUmc), Amsterdam, the Netherlands

**Keywords:** Animal model, Drug development, Forward translation, Reverse translation

## Abstract

Multiple sclerosis (MS) is after trauma the most important neurological disease in young adults, affecting 1 per 1000 individuals. With currently available medications, most of these targeting the immune system, satisfactory results have been obtained in patients with relapsing MS, but these can have serious adverse effects. Moreover, despite some promising developments, such as with B cell targeting therapies or sphingosine-1-phosphate modulating drugs, there still is a high unmet need of safe drugs with broad efficacy in patients with progressive MS. Despite substantial investments and intensive preclinical research, the proportion of promising lead compounds that reaches the approved drug status remains disappointingly low. One cause lies in the poor predictive validity of MS animal models used in the translation of pathogenic mechanisms into safe and effective treatments for the patient. This disturbing situation has raised criticism against the relevance of animal models used in preclinical research and calls for improvement of these models. This publication presents a potentially useful strategy to enhance the predictive validity of MS animal models, namely, to analyze the causes of failure in forward translation (lab to clinic) via reverse translation (clinic to lab). Through this strategy new insights can be gained that can help generate more valid MS models.

## 1 Introduction

Multiple sclerosis (MS) is after trauma the commonest neurological disease in young adults, affecting 1 per 1000 individuals. In the vast majority of patients (80%) the disease initially follows a variable course where episodes of neurological disability (relapse) alternate with complete or partial recovery (remission). This relapsing-remitting phase (RRMS) can last between 5 and 20 years, after which in ± 60% of the cases remissions gradually disappear and symptoms worsen progressively; i.e. secondary progressive (SP) MS [1]. In about 15% of the patients the disease is progressive from the onset, i.e. primary progressive MS. Consensus exists that the neurological symptoms in relapsing MS are caused by autoimmune pathology in the central nervous system (CNS), i.e. brain and spinal cord. The cause of progressive disease is not exactly known, but involves a neurodegenerative process with an important role of resident glia cells, such as microglia and astrocytes [2].

Animal models are important tools in the translational research of MS pathogenesis and treatment [3]. In therapy development their importance is even strategic as they determine the selection of promising drug candidates from the development pipeline. The largest part of current preclinical MS research is based on experimental autoimmune encephalomyelitis (EAE) models in genetically homogeneous strains of specific pathogen-free (SPF)-bred laboratory mice; rats and guinea pigs are nowadays rarely used [4]. However, despite undeniable successes, having yielded 14 approved drugs for relapsing MS [5], the number of drug candidates that fail to reproduce promising effects in EAE models when tested in MS patients remains disappointingly high (>90%). This is not a specific problem for MS. In a recent survey the probability of drug candidates for neurological diseases to reach approval is the lowest among investigated therapeutic areas - i.e. infectious diseases, cancer, respiratory diseases, musculoskeletal diseases, cardiovascular diseases and neurological diseases [6]. This situation is not only frustrating for patients, who are eagerly waiting for safe and effective treatments, but also a waste of valuable resources.

The central theme in this publication is the question which hurdles hinder the translation of scientific concepts on disease mechanisms into safe and effective treatments for MS and how these can be removed. Despite obvious shortcomings there is at this moment no alternative animal model that is equally well characterized, as affordable and as available as the mouse. The question addressed is therefore not whether the mouse should be replaced as elected animal model in drug discovery, but how shortcomings can be identified and improved.

### 1.1 Concise overview of the current MS therapy landscape

**Fig. 1:**
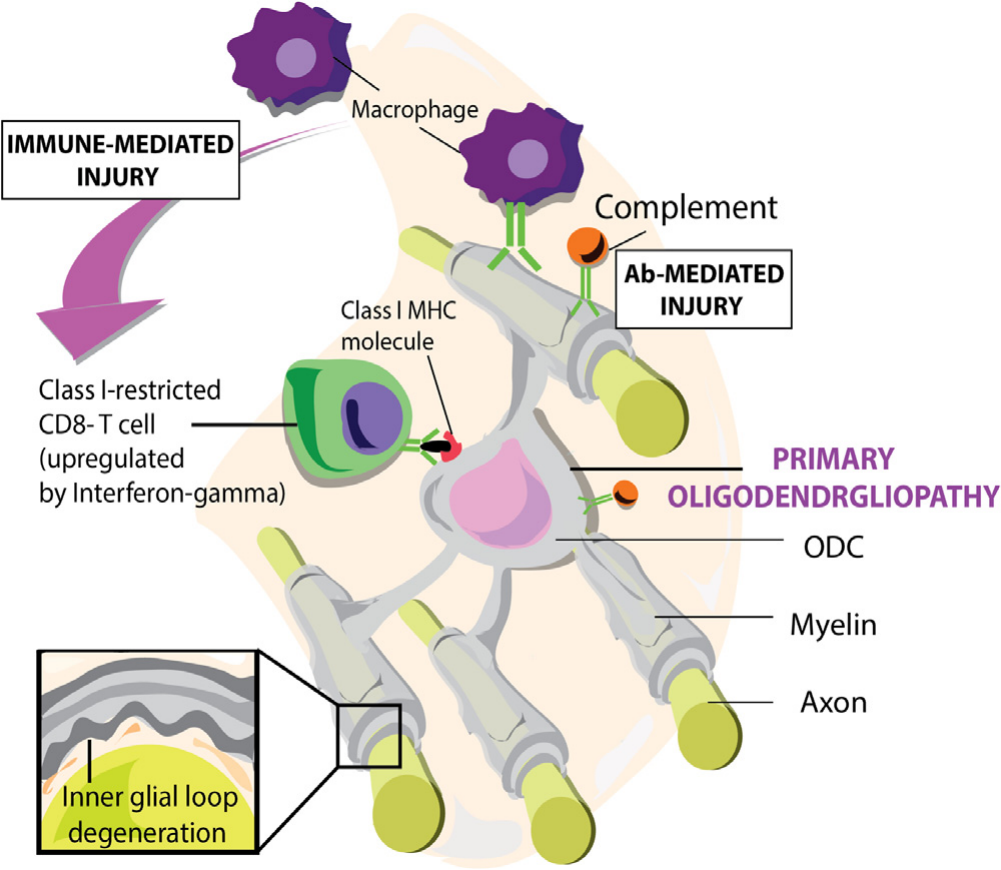
*The autoimmune attack on oligodendrocytes and myelinated axons.* The central process in the pathology of relapsing MS is the infiltration of peripherally induced immune factors. These undertake a combined humoral (antibodies) and cellular (T cells, macrophages) on axon-enwrapping myelin-sheaths and myelin-forming oligodendrocytes. Paradoxically, myelin pathology in MS does not start at the myelin sheath surface, as in EAE models, but at the innermost lamellae that contact the axon. CDC: complement-dependent cytotoxicity, ADCC: antibody-dependent cellular cytotoxicity.

The prevalent MS pathogenic concept and main basis of therapy development is the EAE model. The vast majority of current preclinical MS research data comes from EAE models in a few genetically homogeneous and immunologically naïve (SPF-bred) mouse strains, such as C57BL6, SLJ/J or Biozzi ABH. EAE is usually induced in young adolescent mice (10–12 weeks of age) from genetically susceptible specific pathogen-free (SPF)-bred strains by sensitization against myelin antigens [7]. The ensuing autoimmune pathogenic process involves CNS infiltration of CD4+ *T* helper (Th)1 and Th17 cells that evoke CNS inflammation and opening of the blood brain barrier [8]. By interaction with resident antigen presenting cells (APC), which locally sample antigens, the T cells elicit a cascade of pathophysiological events culminating in a combined cellular (macrophages, cytotoxic T cells) and humoral (antibodies, complement) autoimmune attack on myelinated axons and oligodendrocytes (Fig. 1). Insights into the exact pathogenic role of CD4+ *T* cells comes from a highly elegant adoptive transfer rat EAE model [9]. In such adoptive transfer EAE models T cells from animals with actively induced EAE are transferred via intravenous injection into a naïve major histocompatibility complex (MHC)-compatible recipient.

The first approved drug for MS was β-interferon (IFN). Based on the observation that MS relapses are triggered by an infection, the antiviral cytokine β-IFN was tested in patients with RRMS [10]. The clinical effect was generally modest, although radiological assessment of lesion inflammatory activity with contrast-enhanced magnetic resonance imaging (MRI) showed encouraging beneficial effects. Follow-up studies in the EAE model revealed that the beneficial effect of β-IFN was likely due to its immunomodulatory effects, such as the suppression of IL-12, a cytokine held responsible for skewing T cell differentiation towards a pro-inflammatory phenotype [11].

Despite the limited clinical potency of this drug, it kindled the current dogma that MS is primarily a neuroimmunological condition, which can best be treated via suppression or modulation of immunological processes.

The second drug is Copaxone a.k.a. glatiramer acetate, which was developed in the EAE model [12]. It is a mixture of randomly synthesized polymers from the amino acids l-glutamic acid, l-alanine, l-lysine, and l-tyrosine. The working mechanism involves the modulation of (auto)immune) processes relevant to MS pathogenesis [13]. The drug showed a modest beneficial clinical effect despite marked reduction of contrast-enhancing MRI lesions, provided that it was given early when neurological symptoms are still mild [10].

More potent immunotherapies developed in subsequent years showed beneficial activity in relapsing MS but had limited or no effect in progressive disease. Ablation of the immune system's pathogenic role through treatment with the anti-CD52 monoclonal antibody (Ab) alemtuzumab, the anti-α4β1 integrin mAb natalizumab or reprogramming of the immune system through hematopoietic stem cell transplantation have a profound suppressive effect in relapsing MS. Available data indicate that intervention with these robust medications early in the disease process, may also have an effect on (conversion to) progressive disease [14]. Moreover, drugs preventing the release of activated T cells from the lymphoid organs where they are activated, e.g. the sphingosine-1-P modulator fingolimod, or drugs intervening with sustained activation of T cells, e.g. the IL-2 receptor blocking monoclonal antibody (mAb) daclizumab, mitigate relapsing MS, but have no marked effect on progressive disease. Collectively, these treatments confirm the central pathogenic role of the immune system in relapsing MS, but also indicate that the immune system's pathogenic role may be exchanged for other, currently unknown, pathological processes around the conversion to progressive disease [15].

The sustained immune suppression with these broad-acting interventions can come with serious adverse side-effects. Hence, preclinical researchers worked on the identification of the important cells and factors in the pathogenic process and the development of sophisticated methods to eliminate them functionally or physically. These treatments start from the concept that activation of the autoimmune pathogenic process occurs in peripheral lymphoid organs, where it is accessible for intervention with agents injected into the blood stream.

Intensively investigated targets are:

• *T cells*: the most important T cell subsets in the human immune system are CD4+ *T* helper cells, CD8+ cytotoxic T cells and T regulatory (Treg) cells. In contrast to the central pathogenic of CD4+ *T* cells in mouse EAE models, the dominant T cell type in MS lesions is CD8+ [16]. Unsurprisingly, procedures aimed at mitigating CD4+ *T* cell activation, e.g. with checkpoint inhibitors or antigen-mediated tolerization, are effective in EAE models, but have not been approved for treatment of MS. The current lack of well-characterized mouse MS models in which CD8+ *T* cells have a dominant pathogenic role hampers the development of therapies targeting this subset. Attempts to restrain the autoimmune process by enforcing deficient activity of Treg cells seem successful in EAE [7], but have not (yet) been successful in the clinic.
• *B cells*: the unexpected discovery that depletion of B cells with mAbs binding the pan B cell marker CD20 have a profound and long-lasting effect on relapsing MS [17] and a less albeit beneficial effect in progressive MS [18] has dramatically changed the dominant T-cell centered MS concept. Although the beneficial effect of this treatment is still incompletely understood, it is clear that it does not involve depletion of autoantibody producing plasma cells, which lack CD20 expression, but more likely affects other B cell functions, such as cytokine production or antigen presentation [19].
• *Macrophages*: The pathology of newly forming MS lesions shows besides microglia activation also marked macrophage infiltration, while infiltrating lymphocytes are rare (if at all present). Although the pathogenic role of macrophages in the EAE model and MS has been intensively studied [20], they are not a favorite therapy target in MS.

In summary, despite several successes in the translation of therapies from the mouse EAE model to the MS patients, there is an undeniable large translational gap. This indicates that although in both diseases the immune system has a pathogenic role, the exact immunopathogenic mechanisms may differ substantially.

### 1.2 Why do immunotherapies fail?

The two main reasons for failure in translational drug development are unforeseen toxicity and lack of efficacy [6]. A notorious example of unexpected detrimental effects of an MS drug candidate that showed encouraging effects in the EAE model [21] is the occurrence of fatal PML in a clinical trial of natalizumab, an α4β1-integrin antagonist that prevents leukocyte infiltration into the CNS [22]. An example of an unexpected lack of efficacy is the anti-IL-12p40 antibody ustekinumab; [23]. Obviously, only the animal model can be blamed for the failed translation of an EAE-based pathogenic concept into a therapy. *These failures therefore emphasize that selection of the appropriate animal model for selection of drug candidates is a strategically important decision.*

A set of useful criteria to assess the validity of an animal model for translational research into a given human disease is given in Table 1 [24]. Remarkably, animal models used by scientists in Academia and R&D units of Pharma and Biotech companies are often selected on completely different, more pragmatic criteria, such as low costs, uniform performance between individual animals, stable performance over time and between laboratories, and reproducibility of results. Ironically this is achieved by excluding the very biological factors that determine the risk to develop MS, namely genetic diversity and environmental cues. It can be asked whether the same models would have been chosen when their selection had been based on the validity criteria in Table 1.

**Table 1:** Criteria for the validity assessment of animal models.

**Face validity**	Reflects the degree of similarity in clinical and pathological presentation of the animal model with the human disease.
**Construct validity**	Reflects the degree of similarity in disease mechanisms between the animal model and the human disease.
**Predictive validity**	Reflects the extent to which the animal model correctly predicts the clinical success of an experimental therapy.
**External validity**	Reflects whether the animal model produces comparable results in different research facilities.

Yet another complication that has only rarely been discussed thus far is that the mouse EAE-based pathogenic concept of MS on which therapy developments are projected may inaccurately reflect the situation in MS [16,25,26]. Several lines of evidence show that autoimmunity in MS is triggered by infection of a genetically susceptible host with an (thus far) unidentified microbe, leading to the activation of autoreactive CD4+ *T* cells in peripheral lymphoid organs [27]. The mouse EAE model shows that intervention in this peripheral activation process is feasible and clinically effective, but this may be different in MS. An unbiased study of the scientific literature revealed that it is well possible, if not more likely, that the root cause of MS is not an exogenous factor, but rather a process inside the CNS, i.e. instability of myelinated axons [28]. Building on this new insight we proposed in recent publications that autoimmune pathogenic mechanisms in MS may result from immune hyper-reactivity against critical antigens released from injured axon-myelin complexes [29,30].

In summary, the face validity and/or construct validity of currently used disease models in preclinical research needs substantial improvement to achieve more reliable predictors of clinical success for drug candidates.

### 1.3 How can improvement of EAE models be achieved?

**Fig. 2:**
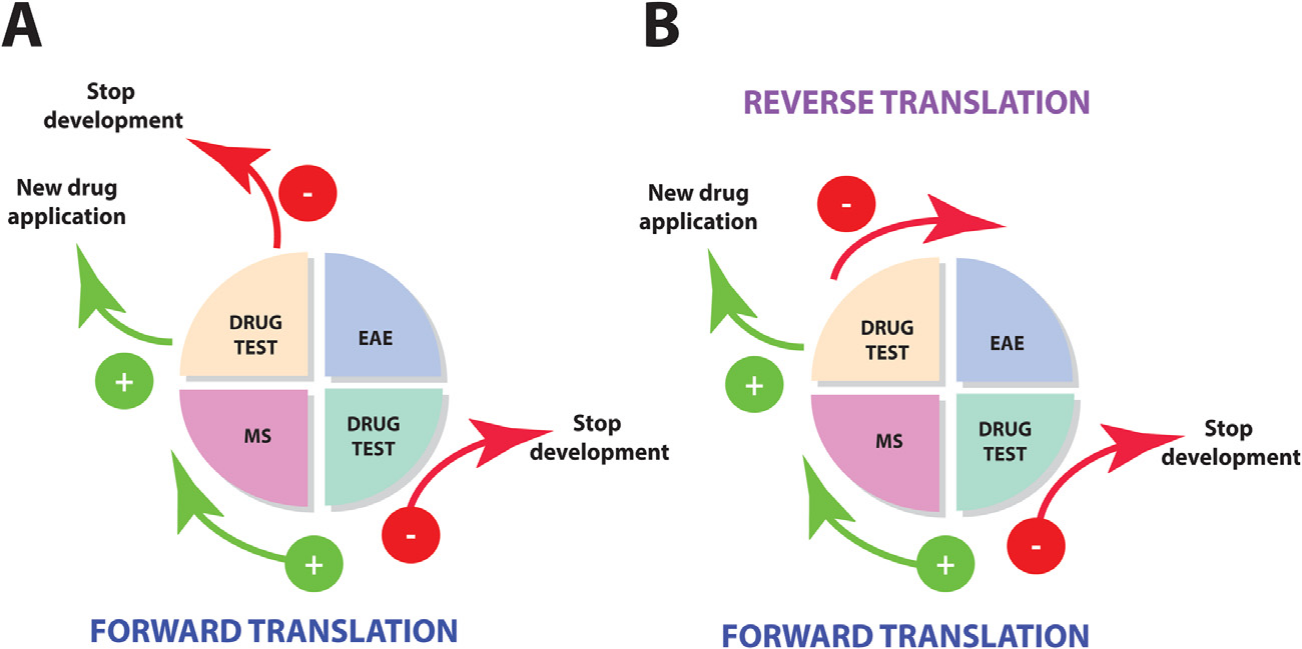
Translational *research as an iterative learning cycle*. A central aim of translational research into pathogenic mechanisms of MS is to convert discoveries in the laboratory into effective treatments for patients. Once a drug shows promising effects in the animal model it is selected for clinical testing (forward translation). When promising effects observed in the animal model are also reproduced in the patient approval from the various regulatory authorities (FDA, EMA, MHRA, PDMA etc.) will be sought. When translation fails, development of the drug is usually stopped (scenario A). We propose that when a negative result is obtained in the clinic, the reason why translation failed should be investigated with the aim to adjust predictive shortcomings of the animal model (reverse translation) (scenario B).

We posit that translational research should not be unidirectional (from lab to clinic), but rather a cyclic, iterative learning process (Fig. 2). When forward translation (from EAE to MS) fails, the cause of failure should be examined through reverse translation (from MS back to EAE), so that factors limiting the predictive validity of the model can be corrected [31,32].

Unfortunately, results from failed clinical trials are often not published. This leaves the false impression that forward translation is often successful and discourages critical reflection on the validity of the used animal model(s). A failure in forward translation, e.g. lack of efficacy or toxicity of a promising new treatment in the patient, essentially implies an expensive lesson that the animal model gave the wrong information. However, when the reason of the failure is not investigated and results of reverse translation are not used for improving the predictive validity of the animal model, the risk that translation fails also in subsequent trials is real.

The selection of a useful animal model for reverse translation experiments should be guided by the validity criteria explained in the previous paragraph (i.e. face, construct, predictive and external validity). The ideal model should be able to bridge the gap between the patient and the mouse EAE model. This is the case in common marmosets, a small-bodied Neotropical primate. Despite an evolutionary distance of 30 Myrs, the immune systems of marmosets and humans are sufficiently alike for testing pharmacological effects of biopharmaceuticals [6]. Importantly, the development of brain pathology in the model can be visualized and quantitated with Magnetic Resonance Imaging techniques that are also used in the clinic. This technology enabled a pseudo-clinical trial design for efficacy testing of new therapeutic agents in the marmoset model [33].

The marmoset EAE model combines essential characteristics of MS and the mouse EAE model [4]. The mouse face of the model comprises an experimentally induced classical mouse-like pathogenic mechanism mediated by CD4+ *T* helper 1 cells, evoking inflammation, and autoantibodies, mediating demyelination (Fig. 1). The human face of the model comprises a novel pathogenic mechanism that emerges from the combined activity of two viral MS risk factors: cytomegalovirus (CMV) [34] and Epstein Barr Virus (EBV) [35]. More specific, B cells infected with the EBV-related γ1-herpesvirus CalHV3 activate CD8+CD56+CD28^null^ effector memory cytotoxic T cells (EM-CTL) that are specific for a mimicry epitope shared by the major capsid protein of CMV (ORF UL86) and the myelin antigen MOG (reviewed in [36]). The combined activity of CD8+ EM-CTL and CalHV3 infected B cells evokes CNS pathology in white and (cortical) grey matter that strikingly resembles MS [37]. Evidence suggests that these T cells are activated by APC presenting MOG released from myelin damaged by the initial CNS attack by CD4+ *T* cells and antibodies [38].

A complication of the marmoset model that is inherent to the outbred nature and the conventional housing conditions is high interindividual variability in the response to EAE induction and to a therapeutic agent [39]. As an example, for a placebo-controlled efficacy assessment of a mAb directed against CD127, the IL-7 receptor, we selected 7 twins from our outbred marmoset colony [40]. We observed that 1 twin did not develop clinically evident EAE during the 22 weeks observation period; 3 twins developed fast progressing EAE and 3 twins developed slow progressing EAE. Intriguingly, the anti-CD127 mAb showed efficacy only in the twins with fast progressing disease. This experiment confirmed the important pathogenic role of anti-MOG CD8+CD56+CD127+ EM T cells in the EAE model observed in earlier studies [41], that is reminiscent to observations in MS by Bielekova et al. [42]. However, as the heterogeneity in clinical and therapeutic response precluded statistical evaluation, we experienced great difficulty in getting this study published. Ironically, a heterogeneous response to treatment is not uncommon in MS clinical trials [18,43] and even a highly successful biological drug like the anti-TNFα mAb infliximab lacks clinical activity in a relevant proportion (30%) of rheumatoid arthritis patients. It remains an open question why in preclinical research statistical significance is often found more important than clinical relevance [44,45].

### 1.4 Lessons learned from reverse translation analysis of failed MS treatments

We believe that clinical trials should be treated as scientific experiments and that failures are equally informative as successes for gaining important insights into the autoimmune pathogenic process in MS.

To illustrate the power of reverse translation analysis, we used the marmoset EAE model for investigating the paradoxical effects of B cell depletion therapies in MS. The reason for testing the clinical effect of the anti-CD20 mAb rituximab (RTX) in relapsing MS was to abolish the production of demyelination-mediating autoantibodies evidenced in the EAE model [46]. However, detailed investigation of the unexpected long-lasting beneficial effect of RTX revealed that this was not mediated by the depletion of the antibody factories (plasma cells), because these do not express CD20. Moreover, serum antibody levels were unaffected in the patients [17]. Adding to the mystery was the observation that treatment with atacicept, a chimeric protein that captures cytokines that B cells need for their survival and differentiation (BLyS, APRIL) worsened lesion activity, although CD20+ *B* cells were depleted from the circulation [47].

In the marmoset model we used a clonal variant of the anti-CD20 mAb ofatumumab (HuMab7D8), which displayed good cross-reactivity with marmoset B cells. As atacicept was not available for research we used mAbs for capturing BLyS and APRIL. In summary, we found that treatment of marmosets with anti-CD20 antibody had a similar profound and lasting clinical effect as ofatumumab in MS, which was caused by profound depletion of all CD20 expressing B cells from the circulation and tissues [48]. We also found that capture of BLyS and APRIL exerted a modest clinical effect despite profound B cell depletion [49]. The explanation of this paradox was that in the latter case a small fraction of CD20+ *B* cells that is infected with the EBV-related γ1-herpesvirus CalHV3 was not depleted [50] [51].

The important lesson from this and subsequent experiments was that the EBV-infected B cell may have a crucial role in ongoing MS [52]. This finding may shed a new light on the still elusive relation of EBV with MS risk [36] and warrants the development of therapies for functional or physical elimination of the cell type (e.g. [53,54]). As the standard SPF-bred laboratory mouse lacks an EBV-related virus this discovery could not have been made in classical mouse EAE models.

In a second set of reverse translation experiments we tested why mAb-mediated neutralization of the pro-inflammatory cytokines IL-12 and IL-23 is clinically effective in mouse EAE models [55], but not in MS patients [23]. Reverse translation analysis of the mAb in marmoset EAE revealed that early treatment with the clinical mAb ustekinumab (anti-IL-12p40) in the marmoset MS model completely abolished disease by inhibiting the mouse EAE-like initiation mechanism that is mediated by Th1/17 cells [56]. However, in a pseudo-clinical trial design where treatment was started once presence of MRI-detectable brain lesions was observed, it was observed that although inflammation and enlargement of cerebral white matter lesions was suppressed, onset of neurological symptoms was only temporarily delayed [57]. The important lessons from these experiments were: 1. That CNS lesion formation and clinical symptoms seem to be induced by different pathogenic mechanisms, and 2. That intervention with the anti-IL-12p40 mAb mitigates the mouse EAE-like EAE activation mechanisms but has no effect on the pathogenic mechanism driving EAE progression.

### 1.5 Future perspectives on translation in drug development

At this moment there is no alternative for the mouse as preclinical MS model that is equally well-characterized, equally affordable and equally available. Although the marmoset EAE model scores high on face- and construct validity, widespread usage is precluded by high costs, the need of specialized facilities for housing and care and increasing ethical constraints. Moreover, despite promising developments in animal-free systems, such as organoids or diseases on a chip, these will not (completely) replace animals in drug discovery in the foreseeable future. Overlooking the many failures in translation, contrasting with only few successes, a critical reflection on EAE in young adolescent immunologically naïve mice as conceptual platform for development of MS therapies is certainly justified. In retrospect, the conclusion is warranted that the mouse EAE model has yielded a broad collection of treatments with which key pathological hallmarks of EAE, autoimmune-driven inflammation and demyelination in the white matter, can be successfully treated. However, these hallmarks represent only part of the complex MS pathology [26].

To enhance the success of translational MS research the conceptual framework on which most therapy development is projected needs adjustment at multiple levels.

*The trigger of autoimmunity*: Overlooking the decades of intensive MS research we deem it highly unlikely that if an exogenous trigger of the MS pathogenic process exists, it could still not have been identified. Notice that for the peripheral demyelinating disease Guillain-Barré syndrome, a causal agent (*Campylobacter jejuni*) has been found already two decades ago, followed in subsequent years by evidences for additional triggers (e.g. *Mycoplasma pneumoniae, Zika virus*) [58]. In MS the debate is still on possible microbe-disease associations without solid proof of causation (guilt by association?).

Mounting evidence in support of an internal trigger should therefore not be ignored. Reports on blistering of myelinated axons [29], microglia nodules [59] and subtle myelin lipid polarity changes [60] document clear abnormalities in the MS brain preceding the autoimmune attack. Discovery of the reason why myelinated axons blister, why myelin constituents are post-translationally modified and why microglia cluster will open new research avenues towards novel therapeutic interventions, which may also be relevant for progressive MS. These therapies should act beyond the usual suspects in the immune system, but rather address the injury that feeds the autoimmune pathogenic mechanisms with (post-translationally modified) antigens [29].

*The concept that autoreactive T cells are naïve*: As discussed elsewhere [61] the question whether the MS trigger comes from outside or inside the body is not trivial as it directly ties in with the question whether autoreactive T cells in MS are naïve or antigen-experienced. It is therefore somewhat naïve to ignore the mounting evidence that the immune systems of a young adolescent SPF-bred laboratory mouse and a human adult differ fundamentally and that these differences can have an enormous impact on the construct validity of animal disease models [62], [63], [64].

Intriguingly, the immune system of MS patients shows evidence of premature aging on the basis of accelerated telomere attrition [65] as well as disrupted neuronal development [66]. A prominent feature of immune aging is the oligoclonal expansion of CD4+ and especially of CD8+ *T* cells with a phenotype suggesting clonal exhaustion (CD28^null^CD161+). This phenomenon has been attributed to replicative stress caused by recurrent exacerbation of latent CMV infection [67]. CD28^null^ T cells in MS and RA are relatively insensitive to T regulatory cells [68], whereas the same cell type in chronic inflammatory lung diseases is resistant to adrenocorticoid hormones due to upregulation of chaperonins and down-regulation of glucocorticoid receptors [69]. This is just one example of a pathogenically important cell type in human autoimmune disease that is completely absent in SPF-bred mice. However, a similar cell type is present in marmosets [70]. The phenotype, specificity and MHC-E restriction of the highly autoaggressive T cells that drive EAE progression in marmosets resemble anti-CMV memory cells [71] that expand in the aging human immune system [72].

*The overvaluation of CD4+ T-cell mediated inflammation as primary therapy target:* The current EAE-inspired research focus on the immune system seems to disregard the strong neurodegenerative component in MS, which is particularly pronounced in the progressive phase but is already present at disease onset [28]. Through horizontal translation, therapies showing positive effects in other neurodegenerative diseases were tested with varying efficacy in progressive MS [5]. Examples of such repurposed treatments, for which the mode of action in the pathogenic process is often not exactly known, are: the tyrosine kinase inhibitors *masitinib* and *evobrutinib*, the phosphodiesterase inhibitor *ibudilast* and the glutamate release inhibitor *riluzole* [42]. However, well-validated animal models for progressive MS in which therapies can be developed are currently lacking.

*The immune status of animal models***.** As most therapies target disease mechanisms, improvement of the construct validity of animal models should have the highest priority. Encouraging attempts are now undertaken to give laboratory mice a more human-like immune system. Several lines of evidence demonstrate the important influence of gut microbiota on human biology and neurological disease and these insights are now transferred to mouse MS models [73], [74], [75], [76], [77]. Another promising development is that by cohousing of SPF-bred laboratory mice with dirty mice from the field or pet shop they acquire a more human-like immune system, with more pronounced activity of CD8+ *T* cells [78].

Yet another interesting development is the accommodation of the crucial pathogenic role of EBV B cells in the MS autoimmune pathogenic process in the EAE model. Horwitz et al. introduced the mouse  γ-herpesvirus 68 strain in his EAE models and documented a similar effect on the disease as we observed in the marmoset EAE model [79]. Moreover, humanized mouse models have been developed in which pathogenic aspects of EBV infection can be investigated [80].

These are all important developments, which will hopefully improve the predictive validity of mouse MS models. If the necessary changes discussed here can be made, the mouse may become a more reliable workhorse for MS therapy development as it has been for decades in basic immunology research. This would be very good news for all stakeholders involved in MS research, not in the least for the patients.

## Declaration of Competing Interest

Bert A. ’t Hart: has served as consultant for EMD Serono

Antonio Luchicchi: no conflicting interests reported

Geert Schenk: no conflicting interests reported

Joep Killestein*:* has speaking relationships with Merck, Biogen, TEVA, Sanofi, Genzyme, Roche and Novartis. AmsterdamUMC, location VUmc, MS Center Amsterdam has received financial support for research activities from Merck, Celgene, Biogen, GlaxoSmithKline, Roche, Teva, Sanofi, Genzyme, and Novartis.

Jeroen J.G. Geurts: has served as a consultant for or received research support from Biogen, Celgene, Genzyme, MedDay, Merck, Novartis and Teva. He is president of the Netherlands organization for health research and innovation.
